# Characterization, Expression Pattern and Antiviral Activities of Mx Gene in Chinese Giant Salamander, *Andrias davidianus*

**DOI:** 10.3390/ijms21062246

**Published:** 2020-03-24

**Authors:** Yanan Liu, Yiqun Li, Yongze Zhou, Nan Jiang, Yuding Fan, Lingbing Zeng

**Affiliations:** 1 National Demonstration Center for Experimental Fisheries Science Education, Shanghai Ocean University, Shanghai 201306, China; yanan6927@163.com; 2 Yangtze River Fisheries Research Institute, Chinese Academy of Fishery Sciences, Wuhan 430223, Chinayongze_527@163.com (Y.Z.); jn851027@yfi.ac.cn (N.J.); fanyd@yfi.ac.cn (Y.F.)

**Keywords:** Chinese giant salamander, *Andrias davidianus*, iridovirus, Mx, expression pattern, antiviral activities

## Abstract

Mx, Myxovirus resistance is an important interferon-stimulated protein that mediates antiviral responses. In this study, the expression and activities of Chinese giant salamander, *Andrias davidianus* Mx gene, AdMx, were investigated. The AdMx cDNA sequence contains an open reading frame (ORF) of 2112 nucleotides, encoding a putative protein of 703 aa. Meanwhile, AdMx possesses the conserved tripartite GTP binding motif and a dynamin family signature. qRT-PCR analysis revealed a broad expression of AdMx in vivo, with the highest expression levels in brain, kidney and spleen. The AdMx expression level in kidney, spleen and muscle significantly increased at 6 h after Chinese giant salamander iridovirus (GSIV) infection and peaked at 48 h, while that in muscle cell line (GSM) was not noticeably up-regulated until 72 h post infection. Additionally, a plasmid expressing AdMx was constructed and transfected into the Chinese giant salamander GSM cells. The virus load and gene copies in AdMx over-expressed cells were significantly reduced compared with those in the control cells. Moreover, compared to the control cells, a lower level of virus major capsid protein (MCP) synthesis in AdMx over-expressed cells was confirmed by Western blot. These results collectively suggest that Mx plays an important antiviral role in the immune responses against GSIV in Chinese giant salamander.

## 1. Introduction

Mx is a kind of antiviral protein induced by type I interferon (IFN), which formed the antiviral mechanism together with other IFN-stimulated proteins during virus infection [1]. Mx is one of the most powerful proteins against pathogen invasion [2] and is highly conserved in vertebrates and invertebrates. Mx proteins mainly contain three important conserved domains: a conserved N-terminal dynamin domain (containing dynamin family signature and tripartite GTP-binding motifs, DYNc), a central interactive domain (CID) mediating self-assembly and a C-terminal GTPase effector domain (GED) (containing a leucine zipper motif) [2]. The feature of Mx GTPases is their antiviral activity against a wide range of viruses by blocking the early stage of viral genome amplification after entering host cells [3].

The Mx gene was first discovered in mouse, and its resistance to orthomyxovirus influenza A was demonstrated [4]. Subsequently, Mx genes were detected in a large number of animal species [4]. In fish, Mx has been investigated in Atlantic salmon, *Salmo salar*) [5], Japanese flounder, *Paralichthys olivaceus* [6], fugu, *Takifugu rubripes* [7] and channel catfish, *Ictalurus punctatus* [8], etc. In invertebrate disk abalone, *Haliotis discus discus*, the Mx gene was cloned and proved to be highly conserved with vertebrate Mxs [9]. Furthermore, different isoforms of Mx genes are found in many species. Two isoforms of Mx genes have been identified in humans [10] and mice [11], seven in zebrafish [12], nine in rainbow trout—*Oncorhynchus mykiss*, and four in European eel—*Anguilla anguilla* [13]. Currently, there are a few reports on Mx in amphibians. In previous research, Over-expression of *Xenopus laevis* type I IFN could significantly increase the expression of Mx to reduce frog virus-3 (FV3) replication both in vitro and in vivo [14]. In Chinese giant salamanders, Mx expression was significantly up-regulated in the IFN over-expressed cells infected by Chinese giant salamander iridovirus (GSIV) [15]. These studies indirectly indicate that the Mx gene of amphibians may have a viral inhibitory effect. However, the function of Mx during antiviral responses in amphibians is far from clear.

The Chinese giant salamander, *Andrias davidianus* is among the rarest animals in China and is classified as a critically endangered species by the International Union for Conservation of Nature and Natural Resources. In recent years, with the rapid development of artificial breeding and cultivation, the diseases of Chinese giant salamander are becoming increasingly serious. Among them, a severe iridovirosis caused by the GSIV has a mortality rate of 100 percent [16]. GSIV belonging to the genus Ranavirus in the family Iridoviridae, was first reported in 2010 in Shanxi Province, and subsequently spread in Shanxi, Sichuan, Jiangxi and Hubei Province. GSIV is a double-stranded DNA virus, whose virus particles are hexagonal and have icosahedral capsids of 130–150 nm in diameter. The virus enters cells through endocytosis or fusion, replicates in the cytoplasm, and progeny viruses aggregate in pseudocrystalline arrays during the late phase of replication. Electron microscopy suggested that GSIV mainly infected the liver, spleen and kidney of Chinese giant salamander [16]. There is still a lack of effective methods to control this disease. Thus, the mechanism of host immune responses against GSIV infection needs to be investigated.

In this study, a Chinese giant salamander Mx (AdMx) was identified and characterized. The expression patterns of AdMx both in vivo and in vitro were profiled and the antiviral effect against GSIV in Chinese giant salamander muscle (GSM) cells was examined. The results of this current study demonstrate that the Mx of Chinese giant salamander can effectively inhibit the replication of GSIV.

## 2. Results

### 2.1. Identification and Molecular Characterization of AdMx

**Figure 1:**
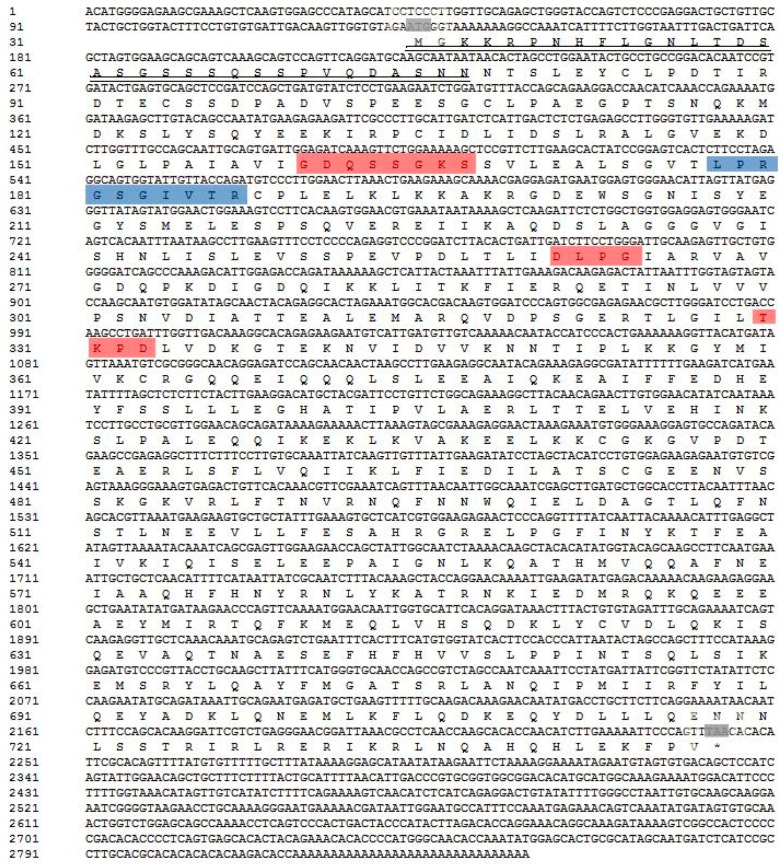
cDNA sequences and the deduced amino acids of AdMx. The gray shades indicate the start codon and stop codon, respectively. Predicted signal peptide is marked with double underline. The asterisk indicates peptide ending. The GTP enzyme dynamic protein superfamily tag sequences are indicated by red shades, and the triplet GTP binding region is indicated by blue shade.

**Figure 2:**
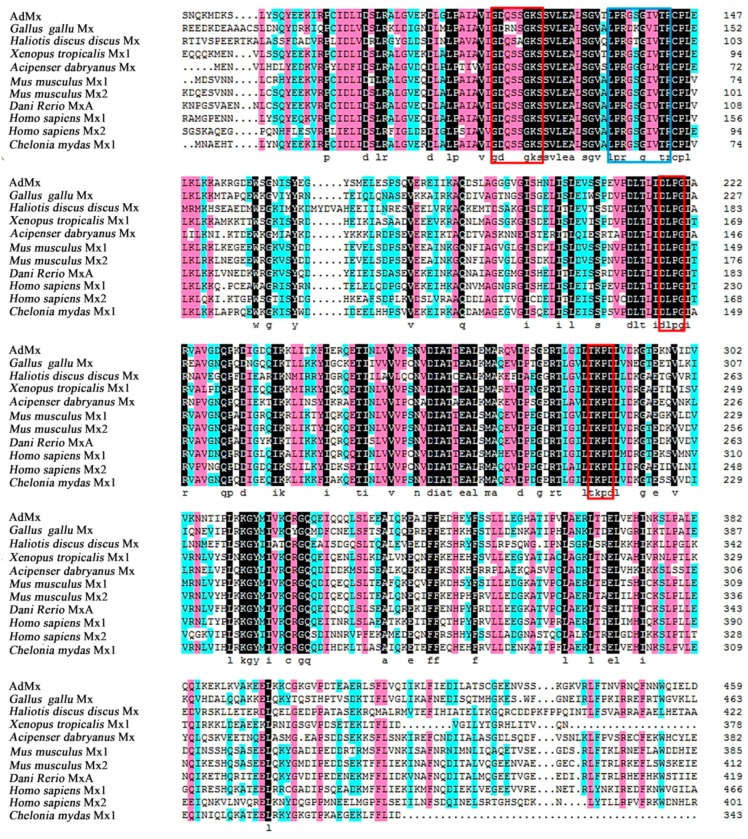
Multi-alignment of AdMx amino acids with other species Mx. Multiple sequence alignment among the amino acids was generated with Clustal W program. Consensus residues are in black, residues that are ≥75% identical among the aligned sequences are in pink, and residues that are ≥50% identical among the aligned sequences are in blue. The GTP enzyme dynamic protein superfamily tag sequences are indicated by red boxes, and the triplet GTP binding region is indicated by blue box. The GenBank accession numbers of the aligned sequences are obtained from National Center of Biotechnology Information (NCBI) and shown as follows: *Gallus gallu* Mx: CAA80686.1; *Haliotis discus discus* Mx: CAA80686.1; *Xenopus tropicalis* Mx1: XP-012813710; *Acipenser dabryanus* Mx: QCB64364; *Mus musculus* Mx1: NP_034976.1; *Mus musculus* Mx2: NP_038634.1; *Dani Rerio* MxA: XP-017206546; *Homo sapiens* Mx1: NP_001171517, *Homo sapiens* Mx2: NP_002454; *Chelonia mydas* Mx1: EM_P31450.

**Figure 3:**
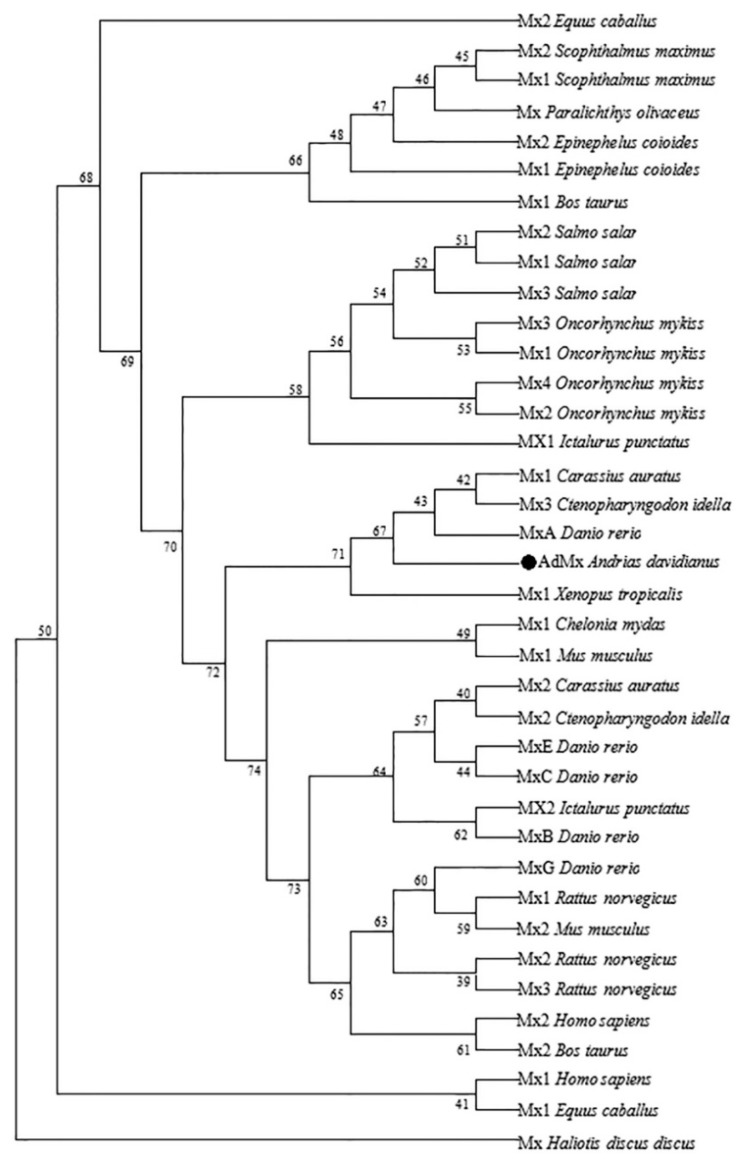
Phylogenetic analysis of AdMx and other Mx proteins. The phylogenetic tree was constructed with MEGA 7.0 software using neighbor-joining method. The AdMx was marked with circle (●). Numbers beside the internal branches indicate bootstrap vales based on 1000 replications. Genebank accession numbers of the selected sequences are obtained from National Center of Biotechnology Information (NCBI) and shown as follows: *Ctenopharyngodon idella* Mx1 ADU33870, Mx2 AAQ95584, Mx3 ADZ44601; *Carassius auratus* Mx1 AAP68828, Mx2 AAP68827; *Danio rerio* MxA NP_891987, MxB Q800G8, MxC NP_001007285, MxE NP_878287, MxG CAD67761; *Epinephelus coioides* Mx1 ABD95979, Mx2 ABD95982; *Ictalurus punctatus* Mx1 Q7T2P0, Mx2 AAY33864; *Mus musculus* Mx1 NP_034976, Mx2 NP_038634; *Oncorhynchus mykiss* Mx1 AAA87839, Mx2 AAC60214, Mx3 AAC60215; *Paralichthys olivaceus* Mx BAC76769; *Scophthalmus maximus* Mx1 AAT57877, Mx2 AAT57878; *Salmo salar* Mx1 AAB40994, Mx2 AAB40995, Mx3 AAB40996; *Haliotis discus discus* Mx ABI53802; *Homo sapiens* Mx1 NP_001171517, Mx2 NP_002454; *Rattus norvegicus* Mx1 NP_001257988, Mx2 NP_599177, Mx3 P18590; *Bos taurus* Mx1 NP_776365, Mx2 NP_776366; *Equus caballus* Mx1 XP_001075961, Mx2 NP_005606216; *Xenopus tropicalis* Mx XP_012813710; *Chelonia mydas* Mx EM_P31450.

The full length of the AdMx cDNA sequences is 2848 bp, with a 5’ UTR (untranslated region) of 132 bp, a 3′ UTR of 604 bp and an open reading frame (ORF) of 2112 bp. AdMx is composed of 703 amino acids residues with a calculated molecular mass of 79.09 kDa and a theoretical isoelectric point of 5.33. Similar to other Mx genes, AdMx possesses a putative hydrophobic signal peptide at the N-terminus (residues 1–33 aa), a GTPase domain containing a triplet GTP-binding region (GDQSSGKS (residues 116–123 aa), DLPG (residues 217–220 aa) and TKPD (residues 286–289 aa)) and GTPase effector domain GED at the C-terminus (610–701 aa) (Figure 1). A dynamin family signature with the sequence of LPRGSGIVTR was also observed in Chinese giant salamander Mx. Sequence alignment indicated that AdMx shared 51%–76% overall sequence identities with the Mx homologues of *Xenopus tropicalis*, *Dani Rerio*, *Acipenser dabryanus*, *Chelonia mydas, Mus musculus*, *Gallus gallus*, *Homo sapiens* and *Haliotis discus discus* (Figure 2). To study the molecular evolution and compare sequence homology, we selected some Mx protein sequences from fish, amphibians, reptiles and mammals in Genbank and constructed a phylogenetic tree (Figure 3). The results showed that AdMx and *Xenopus tropicalis* Mx1 formed a cluster, then formed a branch with the *Danio rerio* MxA, and finally formed a branch with Grass carp, *Ctenopharyngodon idella* Mx3 and Gibel carp, *Carassius auratus* Mx1.

### 2.2. Expression of AdMx in Chinese Giant Salamander Tissues and GSM Cells

**Figure 4:**
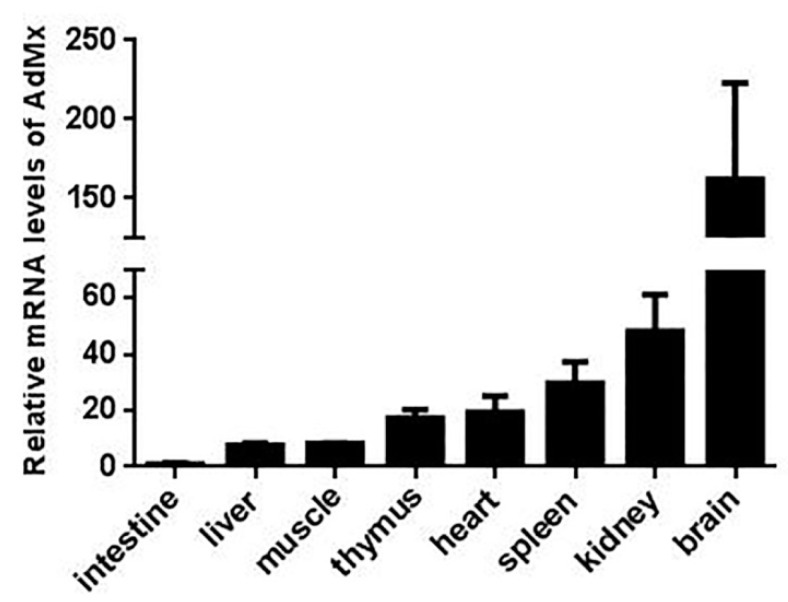
The mRNA expression pattern of AdMx in healthy Chinese giant salamander tissues. AdMx expression in the intestine, liver, muscle, thymus, heart, spleen, kidney and brain was determined by quantitative real time PCR (qRT-PCR) and all data were normalized to β-actin. For convenience of comparison, the expression level in the intestine was set as 1. Error bars indicate the mean ± SD (*n* = 3).

**Figure 5:**
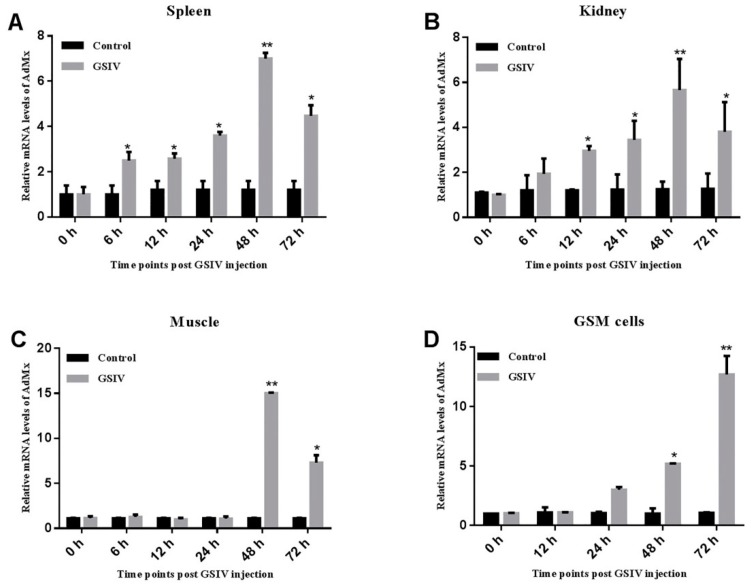
The mRNA expression pattern of AdMx in the spleen (**A**), kidney (**B**), muscle (**C**) and GSM cells (**D**) at indicate times post GSIV infection. The expression level of AdMx was determined by quantitative real time PCR (qRT-PCR) and all data were normalized to β-actin. For the convenience of comparison, the expression level in control at 0 h normalized to β-actin was set as 1. Error bars indicate the mean ± SD (*n* = 3). The asterisks indicate significant difference (** *p* < 0.01, * *p* < 0.05) between treated and control groups.

qRT-PCR analysis indicated that the expression of AdMx was detected in all eight tissues, with the highest expression levels in the brain, intermediate levels in the thymus, heart, spleen and kidney and lowest levels in the intestine, liver and muscle (Figure 4). Following GSIV infection, AdMx expression level in spleen significantly increased at 12 h (2.5-fold), 24 h (3.5-fold) and peaked at 48 h (7-fold) and then decreased slightly at 72 h (4-fold) (Figure 5A). Similarly, in the kidney, AdMx expression level significantly increased at 12 h (3-fold) and 24 h (3.5-fold), and reached a peak at 48 h (6-fold)—higher than the sham-infected controls—and slightly declined at 72 h (4-fold) (Figure 5B). In muscle, AdMx expression levels significantly increased at 48 h (14-fold) and 72 h (7-fold) (Figure 5C). In vitro, a group of GSM cells were infected with GSIV, while the control group was added to the medium as a control. Compared with the control GSM cells, AdMx expression in GSIV-infected cells were significantly up-regulated at 24 h (3-fold) and 48 h (5-fold) and reached the highest expression level at 72 h (13-fold) (Figure 5D).

### 2.3. Evaluation of AdMx Subcellular Localization

**Figure 6:**
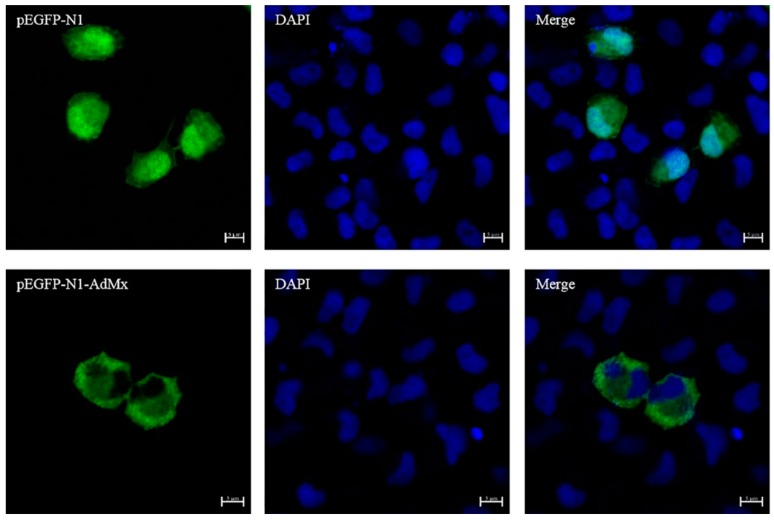
Cytoplasm location of AdMx in transfected GSM cells. GSM cells were transfected with pEGFP-N1-AdMx and stained with DAPI at 48 h post transfection. Scale bar, 5 μm.

The production of recombinant AdMx protein in GSM cells transfected with pEGFP-N1-AdMx plasmid was confirmed by Western blot. The results showed that protein of GSM cells transfected with pEGFP-N1-AdMx plasmid containing AdMx exhibited a molecular weight of 102 kDa, while the control group transfected with pEGFP-N1 plasmid exhibited a molecular weight of 25 kDa (Figure S1). As show in Figure 6, AdMx was mainly expressed in the cytoplasm of transfected cells.

### 2.4. Antiviral effect of AdMx in vitro

**Figure 7:**
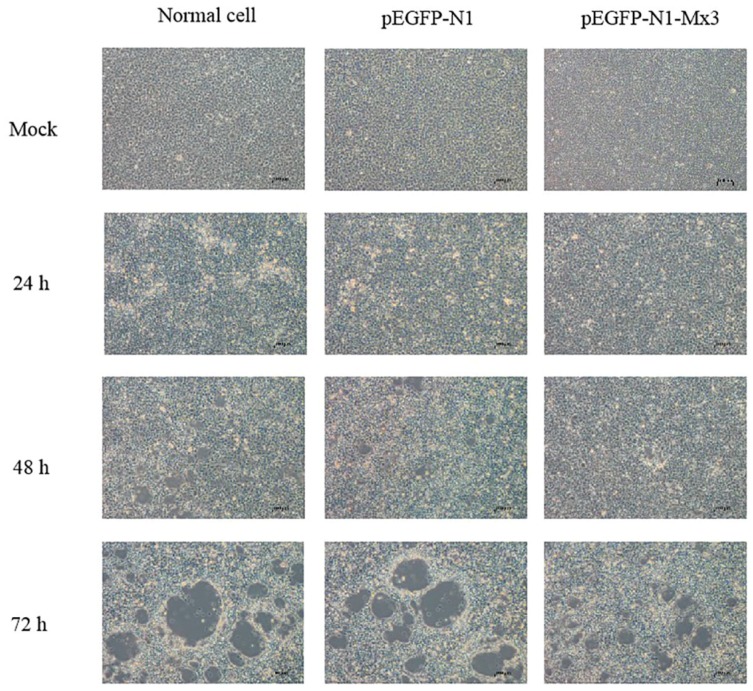
Morphology and cytopathic effect (CPE) of normal GSM cells, pEGFP-N1 transfected GSM cells and pEGFP-N1-AdMx transfected GSM cells at 0 h, 24 h, 48 h and 72 h post GSIV infection. Scar bar, 100 µm.

**Figure 8:**
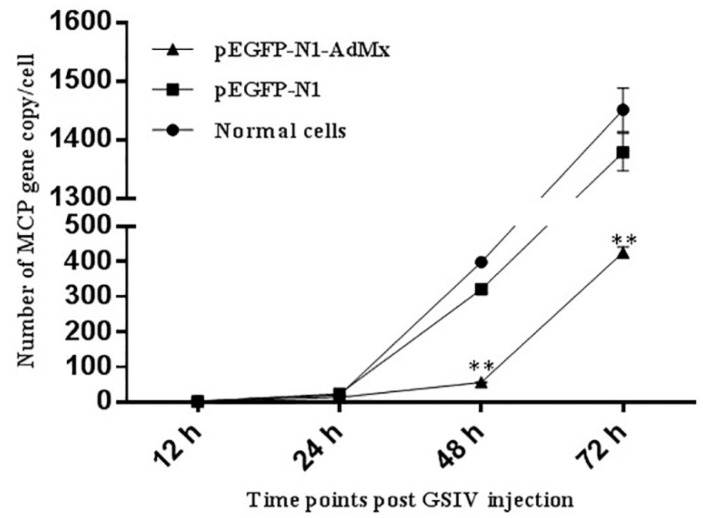
MCP gene copies at indicate times post GSIV infection. MCP gene copies in normal GSM cells, pEGFP-N1 transfected GSM cells and pEGFP-N1-AdMx transfected GSM cells at 12 h, 24 h, 48 h and 72 h post GSIV infection were determined by ddPCR. Error bars indicate the mean ± SD (*n* = 3). The asterisks indicate significant difference (** *p* < 0.01) between treated and control groups.

After infected with GSIV, Cytopathic effect (CPE) of pEGFP-N1-AdMx transfected GSM appeared delayed and more subtle compared to that in cells transfected with the empty vector and non-transfected cells (Figure 7). To confirm the antiviral effect of AdMx on the in vitro viral replication, the gene copies of GSIV major capsid protein (MCP) were analyzed by ddPCR. The results showed that MCP gene copies in the AdMx transfected cells were significantly reduced at 48 h and 72 h relative to that of cells transfected with the empty vector and non-transfected cells (Figure 8).

**Figure 9:**
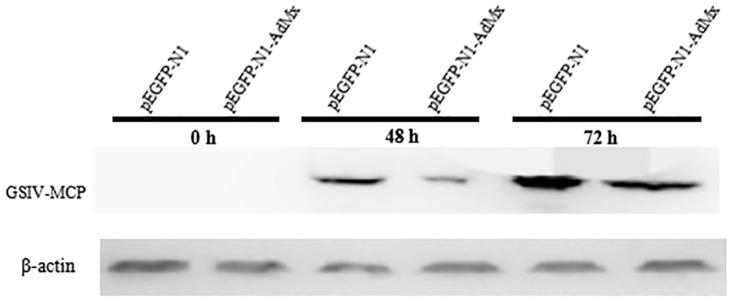
Western blot analysis of MCP protein expression. Western blot was performed on equal amounts of protein harvested from pEGFP-N1 transfected GSM cells and pEGFP-N1-AdMx transfected GSM cells at 0 h, 48 h and 72 h post GSIV infection using anti-MCP monoclonal antibody. β-actin was used as a loading control.

In addition, to quantify the suppression efficiency of AdMx on GSIV protein synthesis, MCP expression was analyzed by Western blot. As shown in Figure 9, viral MCP expression was detected 48 h and 72 h after infection. Compared to the pEGFP-N1-AdMx transfected cells, the MCP expression level was higher in GSM cells transfected with empty vector.

## 3. Discussion

Mx protein is an important component of IFNs-induced antiviral state in many species. It belongs to the class of dynamin-like large GTPases involved in intracellular vesicle trafficking and organelle homeostasis [4]. The GTP-binding motif is important for antiviral activity because GTP binding induces a conformational change of the Mx protein that allows the specific recognition of viral targets [17]. Mutations in the GTP-binding domain may lead to a loss of antiviral activity [18]. Previous studies showed that this motif was highly conserved among species, such as human MxA and MxB [11], mouse Mx1 and Mx2 [12], porcine Mx1 [19], chicken Mx [20], Japanese flounder Mx [21] and Atlantic Halibut Mx [22], etc. In our study, AdMx exhibits typical Mx protein family characteristics, with the conserved tripartite ATP/GTP binding domains (GDQSSGKS/DLPG/TKPD) in the amino-terminal half of the protein. These structural characteristics imply that AdMx is similar to other Mxs in key functional properties. The AdMx amino acid sequences shared 51%–77% identity with other species of Mxs and AdMx, showing it to be closely related to the Mx1 of *Xenopus tropicalis* in phylogenesis, which further indicated that AdMx is a member of the Mx family.

In vertebrates, the expression of Mx was widely detected in various tissues. In ICR mice, Mx1 was expressed in the liver, spleen, kidney, heart and lungs, but not in muscle [23]. Mx1, Mx2 and Mx3 were expressed in 15 tissues of healthy grass carp [24]. In other fishes, the Mx gene is also ubiquitous in different tissues, such as sea bream [25] and Japanese flounder [6]. In the present study, we found that AdMx was expressed ubiquitously in all eight examined tissues, which is similar to the expression pattern in *Cirrhinus mrigala* [26] and *Acipenser dabryanus* [27]. Moreover, AdMx showed a relative high transcript level in the brain, kidney and spleen. The high expression level of Mx in these immune related organs suggests that Mx may be a protective protein against pathogen invasion. In a previous study, the expression of Mx in the head, kidney and blood of *Pseudosciaena crocea* was significantly higher than in that of the control group after 2 days of *Vibrio parahaemolyticus* infection [28]. In gilthead sea bream, the expression of Mx gene in liver increased after *Vibrio alginolyticus* and nodavirus infection [29]. The Mx gene was expressed in the head kidney, spleen, gills, and muscles of turbot infected with Turbot Reddish Body Iridovirus (TRBIV), and reached the highest expression at 1, 4, and 5 day post infection, respectively [30]. After GCRV infection, the expression of Mx gene in the spleen and head kidney tissue of grass carp was significantly higher than that in the control group at 12 h [24]. In the current study, following GSIV infection, AdMx mRNA expression in spleen, kidney and GSM cells showed the same trend of change, with the overall expression increasing initially, and peaking at 48 h post-infection. Intriguingly, Mx gene expression in muscle tissue did not increase until significantly up-regulated at 48 h post-infection. The difference in the expression kinetics of AdMx in vivo and GSM cells following GSIV infection may due to different responses to the same pathogen at the individual and cellular levels.

The antiviral effect of Mx mainly depends on the location of the Mx protein. The Mx gene is usually located in the cytoplasm, and some are also present in the nucleus [31]. Human MxA protein is located in the cytoplasm and has antiviral ability against a wide range of viruses [10]. Mouse Mx2 protein is located in the cytoplasm and is specifically inhibited during the replication phase of the virus [32]. The Mx1 protein of Atlantic salmon is located in the cytoplasm and has antiviral ability against two viruses known as ISAV [33] and IPNV [34]. In the current research, we successfully observed that AdMx is mainly expressed in the cytoplasm of GSM through immunofluorescence assay. Moreover, it is worth noting that previous studies demonstrated that GSIV is assembled and formed in the cytoplasm [16]. These may imply that AdMx plays an antiviral role in the cytoplasm.

At present, the antiviral mechanism of Mx in many species has been successfully studied. Human Mx1 forms oligomeric rings around tubular nucleocapsid structures, thereby inhibiting the transcription and replication of many viruses [35]. The over-expression of the grass carp Mx gene can increase survival of rare minnow—*Gobiocypris rarus*—after GCRV infection [36]. The expression of the barramundi Mx gene is also able to inhibit the proliferation of fish nodavirus and birnavirus [37], and suppresses viral RNA synthesis by interacting with viral RNA-dependent RNA polymerase (RdRp) and redistributing RdRp to the perinuclear area for degradation [38]. Grouper Mx over-expression has an inhibitory effect on nodavirus coat protein and RdRp, which results in reduced viral yields [39]. In the present study, the over-expression of AdMx delayed the appearance of CPE and reduced the MCP gene copies after GSIV infection, which collectively suggested that AdMx could inhibit the replication of GSIV. Furthermore, the reduced GSIV MCP protein expression in the over-expression AdMx GSM cells upon GSIV infection suggested that GSIV replication was inhibited at a protein level. These above results demonstrated that AdMx plays a crucial role in the immune response of giant salamanders to GSIV infection.

To summarize, this study identified an Mx gene from the Chinese giant salamander and examined its cellular localization and antiviral functions. The results in our study indicate that AdMx is a homologue of Mx family, and the over-expression of AdMx exhibits an inhibitory effect on GSIV-caused CPE and the replication of the virus. These results suggest an inhibition role of the Chinese giant salamander Mx in GSIV infection. This study provides new insights for further study on the antiviral mechanisms of the Chinese giant salamander and has important application potential in the development of vaccines and immune adjuvants in the prevention and control of Chinese giant salamander diseases.

## 4. Materials and Methods

### 4.1. Animals, Cells, and Virus

All treatments and procedures for experimental animals were approved by the Animal Care and Use Committee of the Yangtze River Fisheries Research Institute, Chinese Academy of Fishery Sciences.

Chinese giant salamanders (average 190 g in weight) were obtained from our lab. The animals were maintained in tanks at 20 °C for one month and fed with fish every day. The Chinese giant salamander muscle (GSM) cell line was generously provided by Prof. Qi-Ya Zhang (Institute of Hydrobiology, Chinese Academy of Science). GSM cells were maintained at 20 °C in M199 (Hyclone, Logan, UT, USA) supplemented with 10% fetal bovine serum (FBS). The *Epithelioma papilloma cyprini* (EPC) cell line was obtained from the China Center for Type Culture Collection (CCTCC), Wuhan University. GSIV was isolated from the Chinese giant salamander and propagated in EPC cells according to the methods described previously [16].

### 4.2. Cloning of AdMx

Total RNA was extracted from the spleen of Chinese giant salamanders using TRIzol Reagent (Omega Bio-tek, Norcross, GA, USA) and reversely transcribed into cDNA using RevertAid First Strand cDNA Synthesis Kit (Thermo Scientific, Waltham, MA, USA) following the manufacture’s protocol. Based on the transcriptome analysis of Chinese giant salamanders, a pair of primers AdMx-ORF-F (5′-ATGGGTAAAAAAAGGCCAAATC-3′)/AdMx-ORF-R (5′-GTTGTAGAACTTTTTAAGGGTCAA-3′) was designed to obtain ORF sequence. Two pairs of primers were designed according to the obtained ORF sequence: AdMx-3F outer primer (5′- GGTTGCTCAAACAAATGCAGAGTCTGAA-3′)/AdMx-3F inner primer (5′-GATACGTCTGAGGGAACGGATTGAACG-3′) and AdMx-5R inner primer (5′-CCACATTTCTTTAGTTCCTCTTTCGCTAC-3′)/AdMx-5R outer primer (5′-CATCAATGACATTCTTCTCTGTGCCTTTG-3′). Rapid amplification of cDNA ends (RACE) PCR was performed using the SMART RACE cDNA Amplification Kit (Clontech, Mountain View, CA, USA) to identify the 5′ and 3′ untranslated region (UTR) of AdMx gene according to the manufacturer’s instructions separately. All the specific PCR products were purified on 1.5% agarose gels and then inserted into pMD19-T vector (TaKaRa, Taejin, Japan) for sequencing.

### 4.3. Sequence Alignment and Phylogenetic Analysis

The BLAST program at the National Center of Biotechnology Information (available online: http://www.ncbi.nlm.nih.gov/blast/, accessed on 20 October 2019) was used to search sequences. Signal peptide was predicted online using SignalP-4.0 Server (available online: http://www.cbs.dtu.dk/services/SignalP-4.0/, accessed on 21 October 2019). Multiple amino acid sequence alignments were generated with Clustal W (available online: http://www.ebi.ac.uk/Tools/clustalw/, accessed on 23 October 2019). Conserved domains were predicted by SMART (available online: http://smart.embl-heidelberg.de/, accessed on 21 October 2019) and the conserved residues were shaded using DNAMAN (V6). The phylogenetic tree was constructed by MEGA 7.0 using the neighbor-joining (NJ) algorithm.

### 4.4. Detection of AdMx Expression by Quantitative reAl-Time PCR (qRT-PCR)

To examine the expression pattern of the AdMx gene in vivo, three healthy Chinese giant salamanders were sampled after euthanasia with tricaine methanesulfonate MS222 (100 mg/L, Sigma, St. Louis, MO, USA) and their liver, kidney, spleen, heart, intestine, brain, thymus and muscle tissues were collected to RNA extraction. To clarify the effects of GSIV infection on AdMx expression in individual level, thirty-six Chinese giant salamanders were equally and randomly divided into a treatment group and control group. The Chinese giant salamanders of the treatment group were intraperitoneally injected with 200 µL of GSIV (1.0 × 10^7.8^ TCID_50_/mL), while those of the control group were intraperitoneally injected with equal volume of PBS. The spleen, kidney and muscle of three samples were collected from each group at 0, 6, 12, 24, 48 and 72 h post-injection, respectively. Additionally, to examine the expression profiles of the AdMx gene during GSIV infection in vitro, GSM cells (5 × 10^6^ cells/mL) were cultivated in 6-well plates and cultured overnight until a monolayer was formed. Three parallel samples were collected at 0, 12, 24, 48 and 72 h post-GSIV infection (at MOI of 0.01). The control group was treated with same volume of PBS.

Total RNA was isolated using TRIzol reagent (Omega Bio-tek, Norcross, GA, USA) and the residual trace of DNA was removed by DNase I (TaKaRa, Taejin, Japan). Then RNA was transcribed into cDNA as described above. qRT-PCR was performed using the primer AdMx-rqF/AdMx-rqR. The amplification was performed by Rotor-Gene 6000 Real Time PCR system (Qiagen, Dusseldorf, Germany). The qRT-PCR mixture consisted of 2 μL of the diluted cDNA sample, 10 μL Power 2×TB Green Fast qPCR Mix (TaKaRa, Taejin, Japan), 0.8 μL of each primer (10 M) and 6.4 μL H_2_O. The qRT-PCR cycle profile included 1 cycle at 95 °C for 5 min, then 40 cycle at 95 °C for 20 s, 55 °C for 20 s and 72 °C for 20 s. The primers designed for qRT-PCR were as follows: AdMx-rqF: 5′-CCGTTCTTGAAGCACTATCCG-3′, AdMx-rqR: 5′-ACTCCATTCATCTCCTCGTTTTG-3′; β-actin-rqF: 5′-TGAACCCAAAAGCCAACCGAGAAAAGAT-3′, β-actin-rqR: 5′-TACGACCAGAGGCATACAGGGACAGGAC-3′. Relative qRT-PCR gene expression analysis was performed using the 2^−ΔΔ*C*T^ method, with the β-actin gene used as the internal control gene for cDNA normalization [40]. All the experiments were repeated three times.

### 4.5. Construction of AdMx Plasmid and Transfection

The ORF of the AdMx sequence was amplified using LA TaqTM DNA polymerase (TaKaRa, Tokyo, Japan) by PCR with specific primers AdMx-F (5′- CCGGAATTCAATGGGTAAAAAAAGGCCAAATC-3′)/AdMx-R (5′-CCGGAATTCCAACTGGGAATTTTTCAAGATGTTG-3′), containing XhoI and EcoRI sites. The corresponding PCR product and the control plasmid of pEGFP-N1 (Clontech, Mountain View, CA, USA) were digested with XhoI and EcoRI for 5 h. The target fragments encoding the putative mature peptide of the AdMx gene were purified and ligated with T4 DNA ligase, then inserted into the pEGFP-N1 vector and sequenced to verify the reading frame. The recombinant plasmid was designated as pEGFP-N1-AdMx, and the ORF of the AdMx fragment was located between the immediate early promoter of CMV and the EGFP-coding sequences. The recombinant vector pEGFP-N1-AdMx was extracted using the Endo-free Plasmid Mini Kit (OMEGA, Norcross, GA, USA) and then sequenced. Briefly, GSM cells were seeded into 6-well plates at a density of 2 × 10^5^ cells/mL, and cultivated with medium M199 containing 10% FBS for 24 h until the cells achieved approximately 70–80% confluency. Then, 500 μL M199 medium containing 4 μg pEGFPN1-AdMx or the empty vector and 10 μL lipofectamine^TM^ 2000 (Invitrogen, Carlsbad, CA, USA) were introduced into per well according to the manufacturer’s instructions. The medium was changed to fresh M199 containing 10% FBS after 6 h. At 48 h post transfection, cells were harvested and the expression of AdMx was detected via Western blot with mouse anti-EGFP antibody (CST, Danvers, MA, USA).

### 4.6. AdMx Subcellular Localization

To clarify the location of AdMx protein in the cell, GSM cells (2 × 10^5^ cells/mL) were cultivated on glass coverslips in 12-well culture plates for 24 h until the cells reached approximately 70%–80% confluency. Then, 250 μL M199 medium containing 2 μg plasmid and 5 μL lipofectamine^TM^ 2000 (Invitrogen, Carlsbad, CA, USA) were introduced into per well according to the manufacturer’s instructions. The medium was changed to fresh M199 that contained 10% FBS after 6 h. At 48 h post transfection, the cell culture media was removed, and the GSM cells were washed with PBS three times, and then fixed with 4% paraformaldehyde for 20 min. After being washed three times with PBS, the cells were stained with 6-diamidino-2-phenyli-ndole (DAPI) (Solarbio, Beijing, China). Lastly, the coverslips were washed and examined using a fluorescence microscope (Olympus, Tokyo, Japan).

### 4.7. Detection of GSIV Major Capsid Protein (MCP) Gene Copies by Droplet Digital PCR (ddPCR)

GSM cells (2 × 10^5^ cells/mL) were cultivated in 12-well culture plates for 24 h to reach a confluency of 70%–80%. Then, the pEGFPN1-AdMx or an empty vector was transfected as above. At 48 h post-transfection, cells were infected with GSIV at an MOI of 0.01. After 12, 24, 48 and 72 h post-infection, the supernatants containing transgenic cells were harvested and the virus DNA was isolated with Viral DNA Kit (OMEGA, Norcross, GA, USA) according to the manufacturer’s protocol. ddPCR was performed using the primers MCP-ddPCR-F (5′-GCGGTTCTCACACGCAGTC-3′)/MCP-ddPCR-R (5′-ACGGGAGTGACGCAGGTG T-3′). The amplification was performed using the QX200^TM^ Droplet digital PCR^TM^ system (Bio-Rad, Hercules, CA, USA). The ddPCR mixture consisted of 2 μL diluted cDNA sample, 10 μL 2×QX200 ddPCR EvaGreen Supermix (Bio-Rad, Hercules, CA, USA), 0.2 μL of each primer (10 M) and 7.6 μL H_2_O. The ddPCR cycle profile included 1 cycle at 95 °C for 5 min, then 40 cycles at 95 °C for 30 s and 55 °C for 1 min, then 1 cycle at 4 °C for 5 min and 90 °C for 5 min.

### 4.8. Western Blot

The expression of MCP protein was detected by Western blot at 48 h and 72 h after GSIV infection. Protein samples of transfected pEGFP-N1 and pEGFP-N1-AdMx group were prepared as follows: Proteins were resolved by 12% SDS-PAGE and transferred onto a 0.45 nm pore nitrocellulose membrane with the use of Semi-dry blotter (Bio-Rad, Hercules, CA, USA). After being blocked with TBST containing 5% skimmed milk at 37 °C for 1 h, the membranes were respectively incubated with the anti-MCP mouse monoclonal antibody (1:1000) and the anti-β-actin mouse monoclonal antibody for the loading control at 4 °C overnight. Then, the membranes were washed three times with TBST, incubated with alkaline horseradish peroxidase-conjugated anti-mouse IgG at room temperature for 2 h, and finally washed three times with TBST. The membranes were incubated with Clarity^TM^ Western ECL substrate (Bio-Rad, Hercules, CA, USA) for 3 min and then exposed through a gel imaging system (Bio-Rad, Hercules, CA, USA).

### 4.9. Statistical Analysis

The GraphPad Prism 6.0 software (Version X, La Jolla, CA, USA) was used for statistical analysis. All data were expressed as the mean ± standard deviation (SD) and analyzed by one-way analysis of variance (ANOVA) to reveal the statistical significance between samples. A *p* < 0.05 value was considered to be a statistically significant difference, and a *p* < 0.01 value as an extreme difference.

## Supplementary Materials

Supplementary materials can be found at https://www.mdpi.com/1422-0067/21/6/2246/s1. Figure S1. Expression of AdMx in GSM cells by Western blot analysis. Western blot was performed on equal amounts of protein harvested from Normal GSM cells, pEGFP-N1 transfected GSM cells and pEGFP-N1-AdMx transfected GSM cells at 48 h post transfection using anti-EGFP monoclonal antibody. β-actin was used as a loading control.

## Abbreviations

GSIV	Chinese giant salamander iridovirus
GSM	Chinese giant salamander muscle cell line
MOI	Multiplicity of infection
