# Supplementary material for: Characterization, Expression Pattern and Antiviral Activities of Mx Gene in Chinese Giant Salamander, Andrias davidianus
Source: Int J Mol Sci. 2020 Mar 24;21(6):2246. doi: 10.3390/ijms21062246 (PMC7139979; doi:10.3390/ijms21062246)
Supplement: Supplementary file 1 [file ijms-21-02246-s001.pdf]

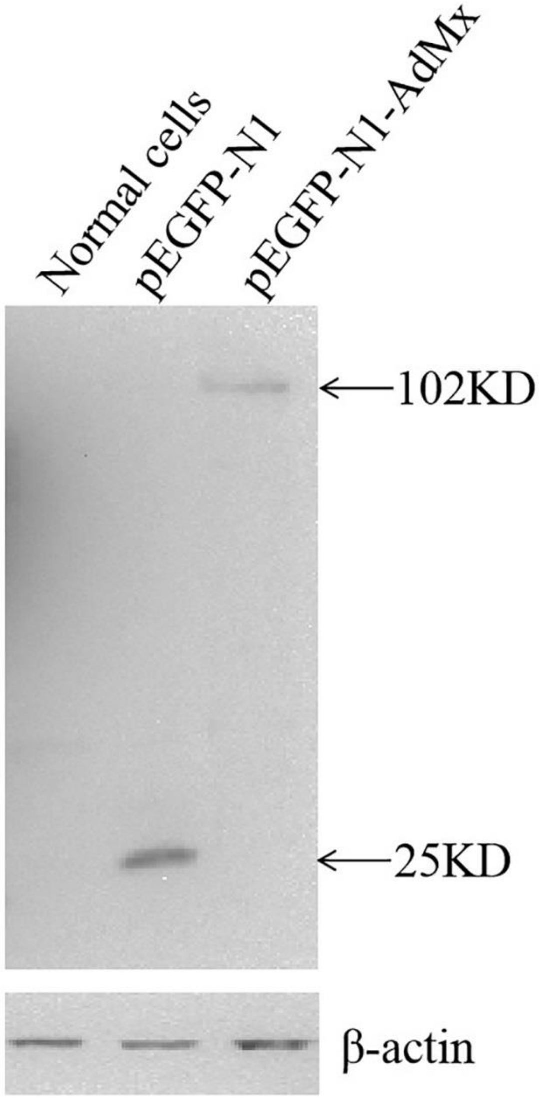

Figure S1. Expression of AdMx in GSM cells by western blot analysis. Western blot was performed on equal amounts of protein harvested from Normal GSM cells, pEGFP-N1 transfected GSM cells and pEGFP-N1-AdMx transfected GSM cells at 48 h post transfection using anti-EGFP monoclonal antibody. β-actin was used as a loading control.
